# Developing Mg-Gd-Dy-Ag-Zn-Zr Alloy with High Strength via Nano-Precipitation

**DOI:** 10.3390/nano13071219

**Published:** 2023-03-29

**Authors:** Jinshu Xie, Jinghuai Zhang, Shujuan Liu, Zihao You, Zhi Zhang, Tengfei Zhao, Xiaobo Zhang, Ruizhi Wu

**Affiliations:** 1 Key Laboratory of Superlight Material and Surface Technology, Ministry of Education, College of Material Science and Chemical Engineering, Harbin Engineering University, Harbin 150001, China; 2 Department of Materials Physics and Chemistry, Harbin Institute of Technology, Harbin 150001, China; 3 Jiangsu Key Laboratory of Advanced Structural Materials and Application Technology, Nanjing Institute of Technology, Nanjing 211167, China

**Keywords:** magnesium alloys, ageing, nano-precipitation, mechanical properties

## Abstract

A high-performance Mg-10Gd-4Dy-1.5Ag-1Zn-0.5Zr (wt.%, EQ142X) alloy was designed by multi-element composite addition in this work, obtaining a high yield strength (~396 MPa) and ultimate tensile strength (~451 MPa) after hot extrusion and ageing. The high strength is mainly related to fine grains and nano-precipitates, especially the latter. *β*′ and *γ*″ nano-precipitation with high fractions are the main strengthening phases, leading to a strengthening increment of ~277 MPa. Moreover, the multi-element alloying in this study promotes the basal-prismatic network strengthening structure, composed of *β*′ nano-precipitation with (1-210) habit planes, *γ*″ nano-precipitation with (0001) habit planes, basal plane stacking faults and 14H-long period stacking ordered phase. In addition, the dislocations and fine grains introduced by the hot-extrusion process not only accelerate the precipitation rate of nanostructure and thus improve the ageing hardening efficiency, but also facilitate the formation of more uniform and finer nano-precipitation. Thus, it is proposed that introducing nano-precipitates network into fine-grained structure is an effective strategy for developing high-strength Mg alloys.

## 1. Introduction

Magnesium (Mg) alloys, the lightest structural alloys with low density of about 1.7–2.0 g cm^−3^, have great application potential in aerospace, military hardware, automotive, 3C (computer, communication and consumer electronic) and other fields [1]. However, low absolute strength seriously affects the wide application of Mg alloys [2]. Numerous R&D efforts have been devoted to developing Mg alloys having high strength [3], and the Mg-Gd series alloys are considered as one of the most potential high-strength Mg alloy systems.

The high strength of the Mg-Gd alloys is mainly ascribed to their excellent precipitation hardening effect generated by the precipitation of the metastable *β*′ nano-precipitation on the prismatic plane [4]. The addition of multiple rare earth (RE) elements may be beneficial to improve the age-hardening behavior and mechanical properties of Mg-Gd alloys [5]. Li et al. [6] designed a new high-strength cast Mg-2Gd-2Nd-2Y-1Ho-1Er-0.5Zn-0.4Zr (wt.%) alloy and found that the multiple RE (RE = Gd, Nd, Y, Ho, Er) alloying instead of single Gd can effectively improve the age-hardening response. In addition, some studies indicated that adding “small” atoms (such as Ag, Zn, Al) to Mg-Gd alloys can significantly improve their age hardening response [7]. Gao and Nie [8] found that the maximum ageing hardness of Mg-Gd-Zr alloy can be greatly improved by controlling the addition of Ag or Zn, and the maximum hardness can be further improved by the combined addition of Ag and Zn. Therefore, according to previous studies, it can be speculated that the synergetic addition of multiple “large” atoms and multiple “small” atoms may increase the strength by introducing more nano-precipitation.

The deformation process is also an important means to improve the properties of Mg alloys. The combination of deformation and ageing may be able to improve age hardening behavior of Mg alloys, obtaining a higher strength increment. On the one hand, the deformation process could introduce a large number of nucleation positions, accelerating the aging process and increasing the number of nano precipitates [9,10]. For example, Li et al. [9] found that simple pre-rolling can improve age hardening response and mechanical properties of Mg-RE alloys by introducing dislocations. Sun et al. [10] designed a high-performance Mg-5.7Gd-1.9Ag wrought alloy via hot rolling and ageing. On the other hand, the hot deformation process can refine grains, and grain refinement would contribute greatly to the strength of Mg alloys due to high Hall-Petch slope [1,11]. Therefore, improving the number of nano precipitates and refining grains by deformation process is an effective way to develop high-performance Mg alloys.

In this work, we designed a novel high-strength Mg alloy by multi-element composite addition, hot deformation, and ageing. Multi-element alloying and dislocations introduced by deformation together promote nano-precipitation. High fractions of nano *γ*″ (ordered hcp, P-62m, a = 0.56 nm, c = 0.44 nm) phase with (0001) habit planes and nano *β*′ (Mg_7_RE, RE = Gd, Dy, orthorhombic, a = 0.65 nm, b = 2.27 nm, c = 0.52 nm) phase with (1-210) habit planes mainly contribute to the age-hardening ability. Moreover, the basal-prismatic network structure formed by 14H-LPSO lamellae, nano-spacing SFs, and nano *γ*″ and nano *β*′ precipitates is favorable for strengthening the alloy. Thus, we propose that introducing multi-precipitates is an effective strategy for developing high-strength Mg alloys.

## 2. Experimental Methods

**Figure 1:**
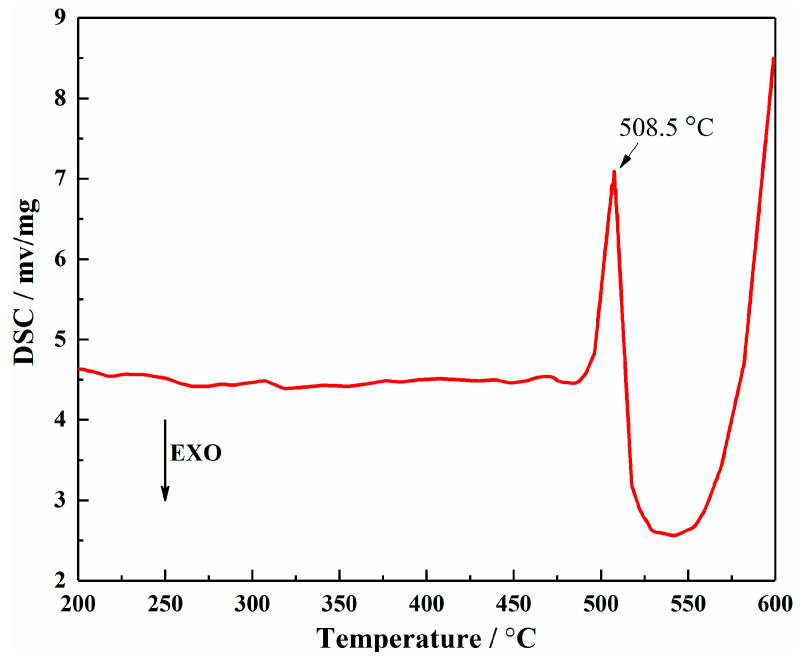
DSC heating curve of the as-cast EQ142X alloy.

The cast Mg-10Gd-4Dy-1.5Ag-1Zn-0.5Zr (wt.%) alloy (EQ142X alloy) was prepared by melting the mixture of pure Mg (99.95 wt.%), pure Ag (99.9 wt.%), pure Zn (99.9% wt.%), Mg-22Gd (wt.%), Mg-21Dy (wt.%), and Mg-30Zr (wt.%) master alloys in an electric resistance furnace under the protection of Ar atmosphere at 750 °C. The actual chemical compositions were analyzed by inductively coupled plasma-atomic emission spectrometry (ICP-AES, Thermo iCAP 7400, Thermo Scientific, Waltham, MA, USA), as listed in Table 1. Then the molten metal was cooled to 730 °C for casting to a cast rod with diameter of 100 mm and height of 700 mm. The differential scanning calorimetry (DSC) test of the cast alloy was done with the heating rate of 5 °C min^−1^ using a Netzsch STA449 apparatus, as shown in Figure 1. Based on the DSC data and multiple test attempts, the as-cast alloy was solution treated at 500 °C for 8.5 h and then quenched by hot water (marked as T4 alloy). The T4 alloy part was extruded by employing an extrusion ratio of 18:1 at 350 °C with a die-exit speed of 9 mm s^−1^ (marked as as-extruded alloy). T4 and as-extruded alloy parts were subjected to ageing treatment at 200 °C, and the peak aged alloys are denoted as T6 and ET6 alloys, respectively. The specific processing processes of all the studied samples are listed in Table 2.

The tensile specimens designed based on GB/T228-2002 standard (gauge length of 20 mm, width of 4 mm, and thickness of 2 mm) were tested using an Instron5869 (Instron Corprration, Canton, MA, USA) with an initial strain rate of 1 × 10^−3^ s^−1^ at room temperature. The direction of tensile samples was consistent with extruded direction (ED), and three samples at least were tested under the same condition to ensure the accuracy of data. The hardness was measured by a Vickers hardness tester at the load of 25 gf and holding time of 15 s. At least twenty points of each sample were tested to maximize the accuracy of the measurements.

The scanning electron microscope (SEM, Quanta200F, FEI company, Hillsboro, OSU, USA) and transmission electron microscope (TEM, Talos F200X G2, FEI company, Hillsboro, OSU, USA) were used to characterize the microstructural features. The samples for SEM were ground using 320#, 800#, 1000#, 1500#, and 3000# SiC sandpapers, and then mechanically polished to a flat mirror. Picric acid solution (1 mL distilled water + 1 mL glacial acetic acid + 7 mL ethanol + 0.42 g picric acid) was used to etch the surface of SEM samples. The samples for electron back scattered diffraction (EBSD, Oxford instruments, Bicester Village, UK) tests were ion milled on a Leica RES101 after mechanical polishing without chemical etching. The EBSD data were collected with a scanning step of 0.3 μm and an accelerating voltage of 20 kV, and they were dealt with using the orientation-imaging microscopy software Azteccrystal (version 2.1, Oxford, UK). The average grain size was measured based on the mean linear intercept method [12]. The samples for TEM were ground to ~40 µm, and then were prepared by ion-milling using a PIPS II system (Gatan 695, Pleasanton, CA, USA) at −30 °C. The TEM results were analyzed using Digital Micrograph software (version 3.7, Pleasanton, CA, USA).

## 3. Results

### 3.1. Mechanical Properties

**Figure 2:**
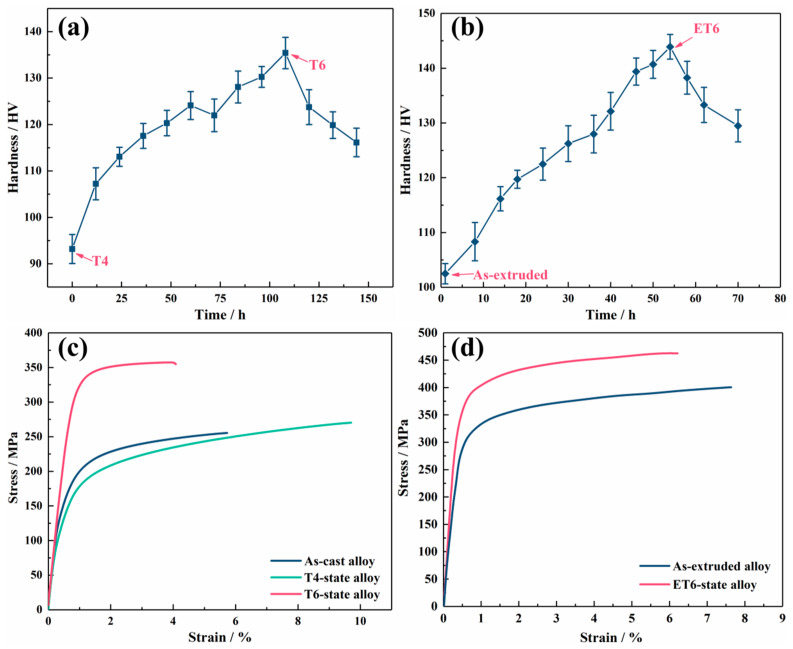
Age hardening curves of (**a**) T4 and (**b**) as-extruded alloys under 200 °C; (**c**,**d**) tensile stress–strain curves of EQ142X alloys with different states.

Figure 2a,b shows the age hardening curves of T4 and as-extruded alloys aged at 200 °C. The T4 alloy can reach the peak value after ageing for more than 108 h, and the hardness increases from the initial ~93 to ~136 HV. It is noted that the peak ageing time of as-extruded alloy (~54 h) is shorter by about half than that of the T4 alloy, and the ET6 alloy obtains a highest peak ageing hardness of ~144 HV. Figure 2c,d shows the tensile stress–strain curves of EQ142X alloys with different states. As expected, the strength of both peak-aged alloys (i.e., T6 and ET6 alloys) is significantly improved. The yield strength (YS) and ultimate tensile strength (UTS) of T6 alloy increase to ~312 MPa and ~356 MPa, while those of ET6 alloy increase to ~396 MPa and ~451 MPa, respectively. They can be considered as the typical “high strength Mg casting alloys” and “high-strength Mg wrought alloys” [5]. Specific mechanical properties of all EQ142X alloys with different states are listed in Table 3.

### 3.2. Initial Microstructure

**Figure 3:**
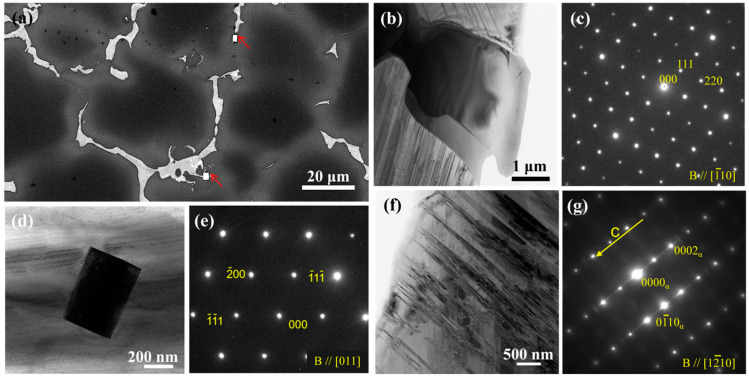
Initial microstructure of the as-cast EQ142X alloy: (**a**) SEM image; (**b**–**g**) bright-filed TEM images and corresponding SEAD patterns. Notes that red arrows marks square-phase particles and yellow arrow represent superstructured lattice.

Figure 3a–g shows the microstructure of the as-cast EQ142X alloy. Based on the mean linear intercept method, the average grain size of the as-cast alloy was measured by multiple SEM images. Figure 3a is a typical location of the as-cast alloy, and the average grain size of the as-cast alloy is ~35 µm. The as-cast alloy is mainly composed of α-Mg equiaxed grains, semi-continuous intermetallic phase, and fine lamellar structure around grain boundaries (GBs). In addition, there are also some bright square-phase particles at the positions marked by red arrows. TEM images and corresponding selected area electron diffraction (SAED) are used to further analyze the three structures mentioned above. The semi-continuous intermetallic phase is identified as Mg_5_(Gd, Dy) with face-centered cubic (fcc) structure (Figure 3b,c), and the lamellar structure with strong streaks between the diffraction spots along the *c*-axis in the SAED pattern is a nano-spacing basal plane stacking faults (SFs) structure (Figure 3f,g). The SAED of square phase in Figure 3e indicates an fcc crystal structure with a = 0.5581 nm. The phase composition is also examined using the energy dispersive X-ray spectrometer, and the fraction of RE atoms is about 85%. Combined with other reports [13,14], it is considered that the square phase is an RE-enriched phase.

**Figure 4:**
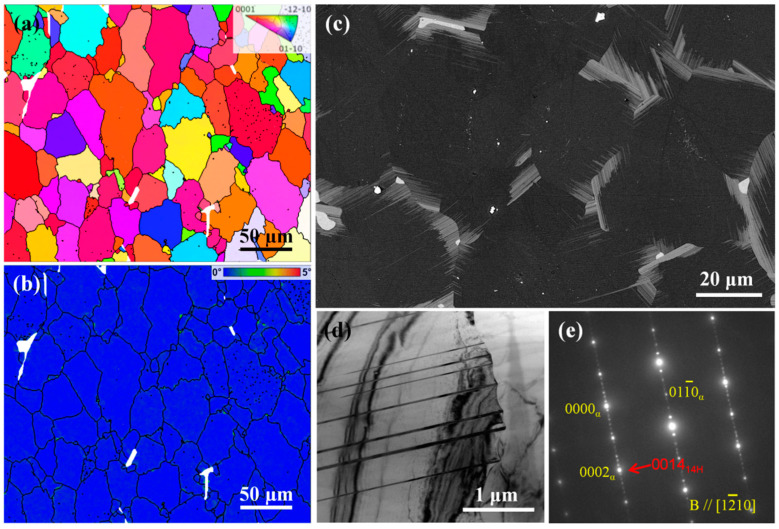
Microstructure of the T4 alloy: (**a**) IPF and (**b**) KAM maps; (**c**) SEM image; (**d**) bright-filed TEM image, and (**e**) corresponding SEAD pattern. Notes that red arrows represent superstructured lattice.

Figure 4 shows the microstructure of T4 alloy. The inversed pole figure (IPF) map and kernel average misorientation (KAM) map in Figure 4a,b show that the average grain size of the alloy changes little (~38 µm) after solution treatment, and the T4 alloy has an extremely low dislocation density. The average KAM value is just/below 0.1°. In general, the dislocation density of well-annealing Mg alloys is ~10^−12^ m^−2^ [15]. SEM observation shows that almost all the Mg_5_(Gd, Dy) phase blocks originally distributed along the GBs disappear after solution treatment, and the lamellar structure around GBs and also the RE-enriched phase still remain in the T4 alloy (Figure 4c). It is worth noting that the lamellar structure at GBs is obviously coarsened (Figure 4c), and the SAED indicates that the lamellae here is 14H-long period stacking ordered (LPSO) structure (stacking sequences: ABABABACBCBCBC [16]) rather than the original SFs (Figure 4e).

**Figure 5:**
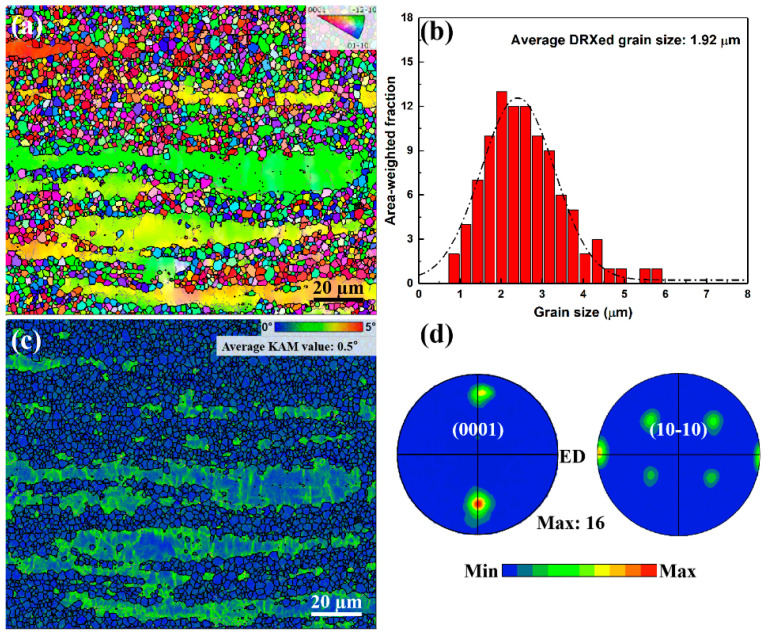
EBSD analyses of the as-extruded alloy: (**a**) IPF map; (**b**) grain size distribution map; (**c**) KAM map; (**d**) (0001) and (10-10) pole figures.

**Figure 6:**
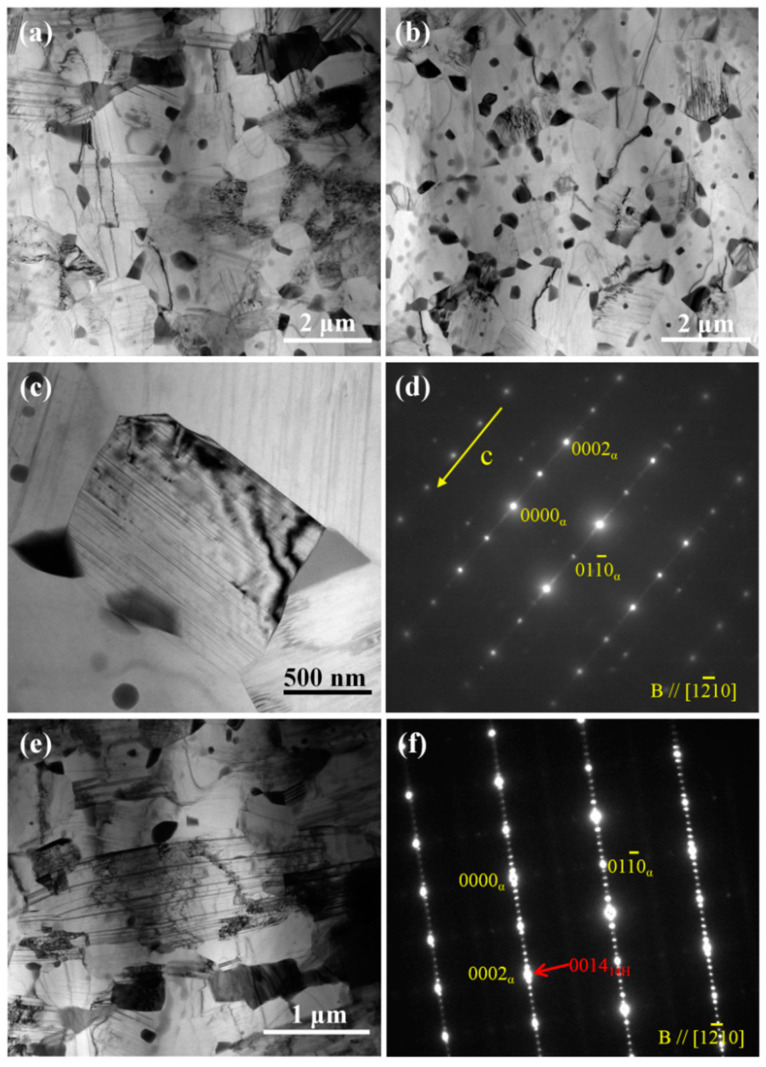
TEM analyses of the as-extruded alloy: (**a**–**c**,**e**) bright-filed TEM images; (**d**) SEAD pattern of lamellar structure within grain; (**f**) SEAD pattern of the block in matrix. Notes that yellow and red arrows represent superstructured lattice.

Figure 5 shows the EBSD analysis of the as-extruded alloy. The IPF map of Figure 5a indicates that the as-extruded alloy has microstructure composed of 66% fine dynamic recrystallized (DRXed) grains and 34% elongated unDRXed grains. The average DRXed grain size of the as-extruded alloy is ~1.9 µm (Figure 5b). The KAM map shows that the unDRXed grains have obvious local stress compared with the DRXed grains, which is related to the higher dislocation density, and the average KAM value is 0.5° (Figure 5c). The as-extruded alloy exhibits a certain basal texture mainly due to the presence of unDRXed grains (Figure 5d). Moreover, the alloy shows a texture having a splitting of basal poles, which may be related to the addition of RE elements [17]. Figure 6a–c shows the TEM images of as-extruded alloys. Hot extrusion induces submicron dynamic precipitation along GBs of DRXed grains. It is well known that Mg-Gd alloys with high alloying are prone to dynamic precipitation during hot deformation processes between 300 and 400 °C, and the dynamic precipitates have been identified as the stable Mg_5_RE phase [18,19]. Moreover, nano-spacing lamellae are also formed within part of the grains during hot extrusion deformation. The streaks of SAED parallel to [0001]_α_ indicate that they are the basal plane SFs (Figure 6d). In addition, the original 14H-LPSO phase is crushed into submicron scale blocks and distributed in the matrix (Figure 6e,f).

### 3.3. Microstructure Evolution after Ageing

**Figure 7:**
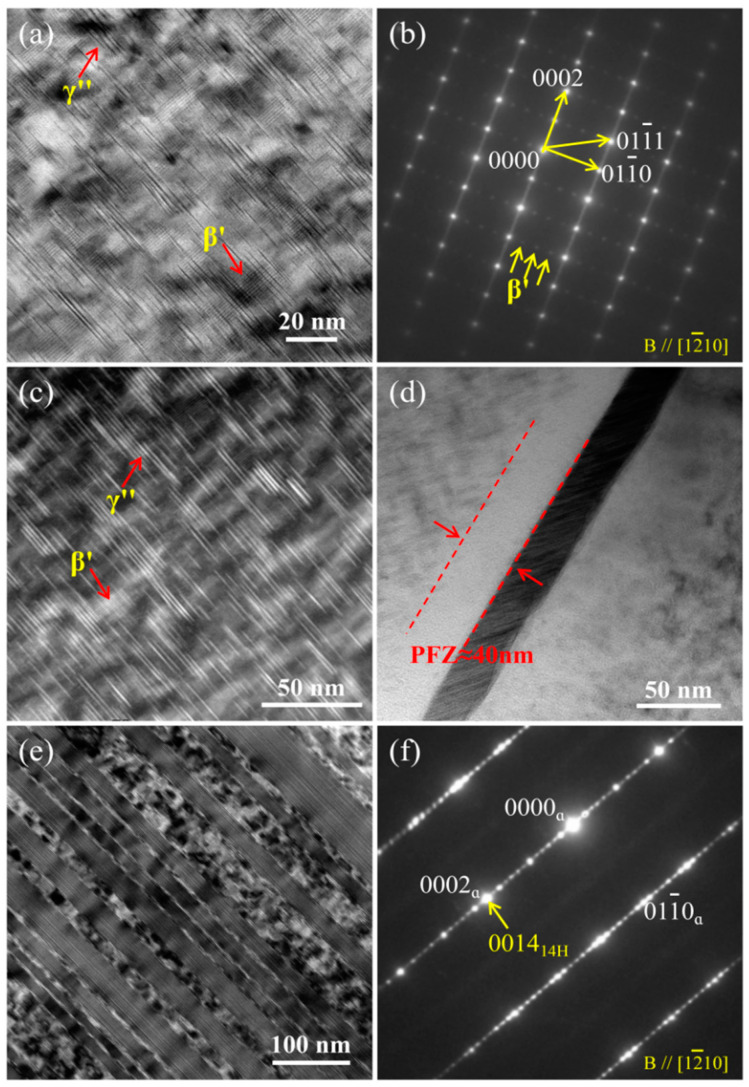
Microstructure of the T6 alloy: (**a**,**b**) and (**e**,**f**) TEM images and corresponding SEAD patterns; (**c**) high-angle annular dark field (HAADF)-STEM image; (**d**) bright-filed TEM image. Notes that yellow arrows represent superstructured lattice.

Figure 7 shows TEM images of the T6 alloy, revealing the main micro-structural features and changes after peak ageing. Figure 7a,c shows that there are two types of nano-precipitates with mutually perpendicular orientations in the alloy matrix. Figure 7b is the SAED pattern along [11-20] corresponding to Figure 7a. In this zone axis, strong streaks parallel to [0001]_α_ are visible (Figure 7b). This, combined with the morphology in Figure 7a and c, indicates the formation of *γ*″ nano-precipitation in the T6 alloy [20]. The additional diffraction spots at 1/4{01$\bar{1}$0}_α_, 2/4{01$\bar{1}$0}_α_, and 3/4{01$\bar{1}$0}_α_ positions are also observed in the SAED pattern (marked by yellow arrows), which correspond to the *β*′ nano-precipitation with elliptical shape [21]. The thickness of the thin foils was measured by convergent-beam electron diffraction (CBED) method [22], and the uniform diameter, the length/thickness of the precipitates, and volume fractions of nano-precipitations were measured using Image-Pro software. The *β*′ nano-precipitates are about 20.94 nm in length and 10.81 nm in width, while the average length and thickness of *γ*″ nano-precipitates are about 35.64 nm and 2 nm. The volume fractions of *β*′ and *γ*″ nano-precipitates are approximately 8% and 13%, respectively. Figure 7d shows that the precipitate-free zone (PFZ) with thickness of 40 nm is formed in the vicinity of GBs in the T6 alloy. In addition, there are still some residual 14H-LPSO lamellae in the T6 alloy, and it can be considered that its density is consistent with that of the T4 alloy nano-precipitation.

**Figure 8:**
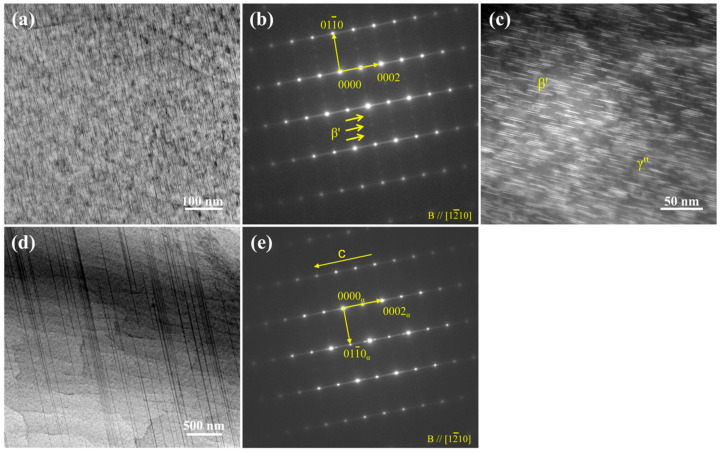
Microstructure of the ET6 alloy: (**a**,**b**) and (**d**,**e**) TEM images along (1-210) direction and corresponding SAED patterns; (**c**) HAADF-STEM image. Notes that yellow arrows represent superstructured lattice.

Figure 8 shows the TEM analysis of ET6 alloy. The bright-field TEM and HAADF-STEM images indicate that two mutually perpendicular nano-precipitations formed in the hot-extrusion alloy after ageing (Figure 8a,c). In the SAED pattern of Figure 8b, both streaks parallel to [0001]_α_ and diffraction spots at ¼{01-10}_α_, 2/4{01-10}_α_, and 3/4{01-10}_α_ are observed. The SAED characteristics combined with the morphological features indicate that the *β*′ precipitates with (1-210) habit planes and *γ*″ precipitates with (0001) habit planes co-exist in the ET6 alloy. The uniform diameter, the length/thickness of the nano-precipitates, and volume fractions of nano-precipitates were measured using Image-Pro software by CBED method. The *β*′ nano-precipitates are about 7.75 nm in length and 7.02 nm in width, and the average length and thickness of *γ*″ nano-precipitates are about 21.82 nm and 2 nm. The volume fractions of *β*′ and *γ*″ nano-precipitates in the ET6 alloy are approximately 5% and 9%, respectively. In addition, the basal plane nano-spacing SFs dynamically formed in the process of hot extrusion still exist after ageing, with almost no change (Figure 8d,e).

## 4. Discussion

In this work, we designed a novel Mg-Gd-Dy-Ag-Zn-Zr alloy by multi-element composite addition and found that ultra-high strength (YS = 396 MPa, UTS = 451 MPa) accompanied by high hardness (~144 HV) can be achieved in the EQ142X alloy by hot-extrusion and ageing treatment. The intrinsic mechanism of the high performance needs further discussion, including nanoprecipitates, fine grains, and dislocations. The YS of the alloy can be described as below:

(1) $$\sigma_{\mathrm{ys}}=\sigma_{0}+\sigma_{\mathrm{GB}}+\sigma_{s}+\sigma_{\mathrm{GND}}+\sigma_{\mathrm{Orowan}}$$

where *σ*_0_ is the friction stress (11 MPa) [23], *σ*_GB_ presents the GB strengthening increment, *σ*_s_ presents the strengthening contribution from solute atoms, *σ*_GND_ presents the strengthening contribution from dislocations, and *σ*_Orowan_ is the potential strengthening increment caused by Orowan mechanism of nanoscale particles.

(a) Multi-precipitates. Firstly, the time to reach the ageing peak value of as-extruded alloy through precipitation is obviously shorter than that of T4 alloy. Based on Cahn’s theory [24] and its revised theory [25], the precipitate nucleation rate is proportional to the volume fraction of dislocations. According to KAM results, the as-extruded alloy shows significantly higher dislocations density (i.e., KAM value) than the T4 alloy, indicating that the as-extruded alloy has a higher precipitate nucleation rate. The higher nucleation rate in the as-extruded alloy promotes the formation of nano-precipitation and makes the size of precipitates smaller, resulting in a more efficient ageing hardening response. Similarly, Li et al. [19] shortened the ageing peak time of Mg-13Gd alloy to 20 h by introducing high-density dislocations through low-temperature extrusion.

Secondly, due to the ingenious composition design of the Mg alloy, a complex basal-prismatic network strengthening structure is produced in the peak aged alloy, and it is made up of nano *γ*″ phase/nano-spacing SFs/14H-LPSO phase with (0001) habit planes and nano *β*′ phase with (1-210) habit planes. Among these phases/structure, the high number density of *γ*″ and *β*′ nano-precipitates are formed during the ageing process. He et al. [26] reported that the decomposition sequence in Mg-Gd-Y-Zr alloys is S.S.S.S → *β*″ (D019) → *β*′ (cbco) → *β*_1_ (fcc) → *β* (fcc). In addition, the addition of Ag into the Mg-Gd-RE alloys is conducive to the formation of *γ*″ nano-precipitates during the ageing process [27]. These two nano-precipitates (*β*′ and *γ*″) would spontaneously be formed and tended to be stable at the ageing temperature of 200 °C in the studied alloys [27]. The addition of Zn is responsible for the formation of 14H-LPSO phase after heat treatment, and also causes the dynamic formation of nano-spacing SFs within grains during hot extrusion processing in the Mg-Gd-Dy alloy. There is almost no change in LPSO phase and nano-spacing SFs after ageing treatment at 200 °C. Yamasaki et al. [28] found that nano-spacing SFs can form in the Mg-Gd-Zn alloy after ageing at 300 °C rather than relatively low temperature of 200 °C, also suggesting that ageing at 200 °C has no effect on nano-spacing SFs.

Thirdly, the hardness of the as-extruded alloy and T4 alloy increases by about 42 HV when reaching peak ageing. The high hardness increase is mainly due to the formation of high number density *γ*″ and *β*′ nano-precipitates in both alloys during ageing. The YS at room temperature should be mainly associated with the basal dislocation slip [4]. The strengthening increment caused by *β*′ and *γ*″ nano-precipitates can be calculated by the following formula [4]:

(2) $$\Delta \tau_{basal}^{\gamma \prime \prime /\beta \prime }=\frac{Gb}{2\pi \sqrt{1-\nu }(\frac{0.953}{\sqrt{f_{p}}}-1)d_{t}}ln\frac{d_{t}}{b}$$

where *ν* is the Poisson’s ratio (~0.35 for Mg), *G* is the shear modulus (~16,600 MPa for Mg), *b* is the Burgers vector of the gliding dislocations (~0.32 nm for <a> dislocations dominated Mg alloys), and *f_p_* and *d*_t_ are the volume fraction and uniform diameter of the precipitates, respectively. The *f_p_* can be calculated by following formula [29]:

(3) $$f_{p}=N\pi d_{t}{}^{2}t/4$$

where *N* is the number density of the precipitates, *t* is the length/thickness of the precipitates. The Δ*τ_basal_^γ^^″^*and Δ*τ_basal_^β^*^′^ are calculated to be ~132 MPa and ~145 MPa in the ET6 alloy, and ~90 MPa and ~139 MPa in the T6 alloy, respectively. Therefore, *σ*_Orowan_ is about 277 MPa in the ET6 alloy, and 229 MPa in the T6 alloy. The *β*′ nano-precipitates with (1-210) habit planes should hinder the basal dislocation movement to the greatest extent and have better strengthening effect. The strengthening effect differences mainly come from the fraction of nano-precipitates. In addition, it is worth noting that although the proportion of nano-precipitates in the ET6 alloy is lower than that in the T6 alloy, the strengthening increment after ageing is still high. It is inevitable that many nano-precipitates, such as Mg_5_(Gd, Dy) and SFs, would be precipitated during hot extrusion, which would reduce the subsequent ageing precipitation of *β*′ and *γ*″ to a certain extent. However, due to the extrusion processing, there are more dislocations in the as-extruded alloy, which results in higher number density and smaller size of nano-precipitates during ageing. In addition, the PFZs are only observed in the T6 alloy but not in the ET6 alloy, indicating that fine grains inhibit PFZ, and the as-extruded alloy has more uniform nano-precipitations.

Finally, the prismatic *β*′ nano-precipitates and basal *γ*″/SFs/14H-LPSO precipitates form basal-prismatic network strengthening structure, offering stronger barrier to dislocation slipping and twin nucleating than monotonous prismatic or basal phases and obtain a higher strengthening increment [4]. Zhang et al. [30] also found that a similar nano-precipitation network structure in Mg-Sm-Yb-Zn alloy can provide the alloy with significantly higher heat resistance than a single precipitated phase.

(b) Solid solution strengthening. The solid solution strengthening is not negligible for highly-alloyed Mg alloys. The increment caused by solid solution element can be calculated as follows [31]:

(4) $$\sigma_{s}=\frac{3.1\varepsilon GC^{0.5}}{700}$$

where *ε* is an experimental constant, equal to 0.74 for Mg-Gd series alloy [31], and *C* is the solute concentration in atomic percentage in Mg matrix. The solute concentration is equal to 0.58 at.% for the T6 alloy, and 0.20 at.% for the ET6 alloy, which is obtained by TEM-EDS. The increment caused by solid solution element is about 42 MPa in the T6 alloy, and 27 MPa in the ET6 alloy. Yang et al. [31] also obtained a solute concentration of approximately 0.61 at.% in the aged Mg-9Gd-3Y-0.6Zn-0.5Zr (wt.%) alloy.

(c) Fine grains. The average DRXed grain size of the as-extruded alloy is ~1.9 µm and fine grains would lead to a high strength increment. Fine DRXed grain size for as-extruded and ET6 alloys would contribute to the high strength based on the Hall–Petch relationship and the increment in strength caused by GBs can be given as follows [1].

(5) $$\sigma_{\mathrm{GB}}={kd}_{\mathrm{GB}}{}^{-1/2}$$

where the *k* is the Hall–Petch slope. He et al. [32] reported that *k* is about 164 MPa μm^−1/2^ for as-extruded and peak-aged Mg-Gd-Y-Zr alloys. This *k* value can be used to evaluate the GB strengthening effect in the present study, ignoring the minor difference in composition. When the ageing temperature is 200 °C, the grain growth driving force of the EQ142X alloy with high RE alloying is very small, and it can be considered that the grain size changes little during the ageing process. Therefore, the strengthening increment caused by GBs is about 118 MPa in as-extruded and ET6 alloys, and about 28 MPa in T4 and T6 alloys, respectively. In addition, the fraction of DRXed grains is about 66% in the as-extruded alloy, the strengthening increment caused by DRXed grains is about 78 MPa.

(d) Dislocations density. The contribution of dislocations for the YS can be calculated by the following equation [15]:

(6) $$\rho_{\mathrm{GND}}=\frac{2\bar{\mathrm{KAM}}}{ub}$$

(7) $$\sigma_{\mathrm{GND}}=\alpha Gb\sqrt{\rho_{\mathrm{GND}}}$$

where *α* is a constant (~0.50), *u* is equal to the scanning step length of EBSD, and *b* is the Burgers vector of the gliding dislocations (~0.32 nm for Mg alloys). The density of the geometrically necessary dislocations (GNDs) can be calculated by using the EBSD technology from KAM map, and the *ρ*_GND_ of the as-extruded alloy is about 2.53 × 10^14^ m^−2^. The YS increment caused by dislocations in the as-extruded alloy is about 43 MPa. Dislocations strengthening mainly comes from 34% elongated unDRXed grains, therefore, the strengthening increment of GNDs is about 15 MPa. Since the dislocation density must be slightly lower than that of the as-extruded alloy after ageing, the YS increment caused by dislocations in the ET6 alloy would be below 15 MPa. Therefore, dislocations contribute less to the strength of the ET6 alloy, and the strengthening caused by dislocations in as-cast, T4, and T6 alloys is almost negligible due to lower dislocation density.

The calculated strength values of high-strength T6 and ET6 alloys basically correspond to the experimental values (Table 4), and the minute differences may be related to the selection of *k* value and basal-prismatic network structure. The *k* value is related to the grain size, texture, and alloy composition; therefore, it is difficult to obtain a completely consistent *k* value. In addition, the basal-prismatic network structure would offer additional reinforcement, which cannot be quantified.

## 5. Conclusions

In this work, we prepared a novel Mg-Gd-Dy-Ag-Zn-Zr alloy by multi-element addition. After the ageing process, the hardness and strength of peak-aged alloys are significantly improved. Especially, high strength (YS = 396 MPa, UTS = 451 MPa) accompanied by high hardness (~144 HV) can be achieved in the ET6 alloy. The main conclusions are listed as follows.

(a) The as-cast alloy is mainly composed of α-Mg equiaxed grains, semi-continuous Mg_5_(Dy,Gd) phase, and nano-spacing SFs at GBs. The microstructure of the as-extruded alloy is characterized by the formation of fine DRXed grains, the dynamic formation of basal plane nano-spacing SFs within grains, and the original 14H-LPSO phase distributed in the matrix.
(b) The YS increments caused by nano-precipitates are 277 MPa in the ET6 alloy, which mainly comes from the *β*′ nano-precipitates with (1-210) habit planes and *γ*″ nano-precipitates with (0001) habit planes. The YS increments caused by DRXed grains is about 78 MPa in the ET6 alloy, which also contributes to the high strength.
(c) The addition of multiple elements (“large” atoms: Gd, Dy; “small” atoms: Zn, Ag) results in multiple aging precipitation sequences in the alloy, promoting the formation of basal-prismatic nano-precipitates network structure in the studied peak-aged alloys. The network structure consists of *β*′ nano-precipitates with (1-210) habit planes, *γ*″ nano-precipitates with (0001) habit planes, basal plane SFs, and 14H-LPSO phase.

## Figures and Tables

**Table 1:** Chemical compositions of the alloys determined by ICP-AES.

Alloy	Actual Composition (wt.%)					
	Mg	Gd	Dy	Ag	Zn	Zr
EQ142X alloy	Balance	9.35 ± 0.13	4.15 ± 0.05	1.45 ± 0.03	1.21 ± 0.03	0.48 ± 0.02

**Table 2:** Specific processing process of the studied samples.

Samples	Processing Technology
As-cast	Pouring at 730 °C
T4	Solution treated at 500 °C for 8.5 h and then quenched by hot water
T6	Peak-aged condition of T4 alloy aged at 200 °C
As-extruded	The T4 alloy was extruded by employing an extrusion ratio of 18:1 at 350 °C with a die-exit speed of 9 mm s^−1^
ET6	Peak-aged condition of as-extruded alloy aged at 200 °C

**Table 3:** Mechanical properties of EQ142X alloys with different states.

Alloys	YS (MPa)	UTS (MPa)	Elongation (%)	Hardness (HV)
As-cast	204 ± 3	255 ± 2	5.7 ± 0.8	-
T4	197 ± 3	270 ± 3	9.7 ± 1.2	93 ± 5
T6	312 ± 4	356 ± 2	4.5 ± 0.5	136 ± 5
As-extruded	352 ± 4	403 ± 3	7.9 ± 0.6	103 ± 4
ET6	396 ± 4	451 ± 5	6.4 ± 0.8	144 ± 5

**Table 4:** The calculated strengthening contributions (MPa) in the T6 and ET6 alloys.

Samples	Grain Boundary Strengthening (MPa)	Dislocations Strengthening (MPa)	Orowan Strengthening (MPa)	Solution Strengthening (MPa)	YS Predicted Result (MPa)	YS Experimental Results (MPa)
T6	28	-	229	42	310	312
ET6	78	15	277	24	405	396

## Data Availability

The raw/processed data required to reproduce these findings cannot be shared at this time as the data also form part of an ongoing study.
